# Impact of preterm birth on kidney health and development

**DOI:** 10.3389/fmed.2024.1363097

**Published:** 2024-03-27

**Authors:** Sara Deffrennes, Maissa Rayyan, Tom Fidlers, Lambertus van den Heuvel, Elena Levtchenko, Fanny Oliveira Arcolino

**Affiliations:** ^1^Department of Development and Regeneration, Katholieke Universiteit Leuven, Leuven, Belgium; ^2^Department of Nephrology, Dialysis and Renal Transplantation, University Hospitals Leuven, Leuven, Belgium; ^3^Neonatal Intensive Care Unit, University Hospitals Leuven, Leuven, Belgium; ^4^Department of Gynecologic Oncology, Oscar Lambret Cancer Center, Lille, France; ^5^Department of Pediatric Nephrology, Radboud University Medical Center, Nijmegen, Netherlands; ^6^Department of Pediatric Nephrology, Emma Children’s Hospital, Amsterdam University Medical Centers, Amsterdam, Netherlands; ^7^Emma Center for Personalized Medicine, Amsterdam University Medical Centers, Amsterdam, Netherlands

**Keywords:** preterm birth, nephrogenesis, premature infant, nephron, chronic kidney disease, acute kidney injury

## Abstract

Preterm birth, defined as birth before the gestational age of 37 weeks, affects 11% of the newborns worldwide. While extensive research has focused on the immediate complications associated with prematurity, emerging evidence suggests a link between prematurity and the development of kidney disease later in life. It has been demonstrated that the normal course of kidney development is interrupted in infants born prematurely, causing an overall decrease in functional nephrons. Yet, the pathogenesis leading to the alterations in kidney development and the subsequent pathophysiological consequences causing kidney disease on the long-term are incompletely understood. In the present review, we discuss the current knowledge on nephrogenesis and how this process is affected in prematurity. We further discuss the epidemiological evidence and experimental data demonstrating the increased risk of kidney disease in these individuals and highlight important knowledge gaps. Importantly, understanding the intricate interplay between prematurity, abnormal kidney development, and the long-term risk of kidney disease is crucial for implementing effective preventive and therapeutic strategies.

## 1 Introduction

In 1988, Brenner and colleagues proposed that a reduction in functional nephron number, either acquired or inherited, might predispose for hypertension and kidney disease in adult age (1). The authors hypothesized that a reduction in whole kidney glomerular surface area would limit the sodium excretory capacity, thereby enhancing susceptibility to hypertension, which would in itself result in glomerular injury and further loss of functional nephron numbers. Evidence in support of this hypothesis was provided by a study showing that the number of glomeruli is lower in the kidneys of patients with hypertension than in the kidneys of matched normotensive controls (2). At about the same time, the epidemiologist David Barker proposed the fetal origins hypothesis of adult disease, now known as the developmental origins of health and disease hypothesis (DOHaD), which originally postulated that suboptimal events during prenatal development increase the risk of cardiovascular disease in adulthood (3, 4). This conclusion was first drawn when Barker et al. found that early death secondary to coronary artery disease was inversely related to weight at birth (5). The range of adult chronic diseases linked to fetal and early postnatal origins has now expanded to include cardiovascular disease, renal disease, metabolic syndrome and diabetes (6, 7).

Every year, *circa* 15 million newborns are born prematurely, accounting for approximately 11% of births worldwide (8, 9). Over recent decades, survival of prematurely born infants has improved markedly due to the advances in perinatal care. Those born as early as 25 weeks of gestation are now reported to have an 80% chance of survival and even infants born as early as 22 weeks of gestation with birth weights close to 500 g now have a survival chance of about 28% (10–12). Consequently, late manifestations of prematurity-associated complications have become evident and clinically relevant. The spectrum of diseases associated with preterm birth are presumed to arise due to the interruption of normal organogenesis of multiple organ systems, including that of the lungs, heart, eyes, brain, digestive tract and the kidneys. Following Brenner and Barker’s hypotheses, a surge in epidemiological and experimental research has demonstrated an association between preterm birth and development of kidney disease in adulthood (13). Chronic kidney disease (CKD) affects about 10% of the world’s population and therefore imposes a major burden to health systems worldwide. With the current increase in CKD prevalence, it is projected that kidney failure will be the fifth leading cause of death by 2040 (14). Since it has been reported that prematurity can increase this baseline risk of CKD by two-to three times (15), improved understanding on how prematurity affects kidney development and health is crucial to develop new therapeutic and diagnostic tools.

The present review aims at describing the evidence linking prematurity with abnormal kidney development and subsequent development of kidney disease. We discuss the epidemiology of preterm birth and its risk factors. We further describe the current knowledge on human kidney development and highlight how preterm birth alters this process. We then review the observational data supporting the link between preterm birth and kidney diseases and the potential mechanistic factors underlying this link. To conclude, we identify important knowledge gaps that need to be addressed to improve the management of patients born preterm with regard to their short-and long-term renal health.

## 2 Preterm birth: definition, epidemiology, causes and risk factors

**Figure 1:**
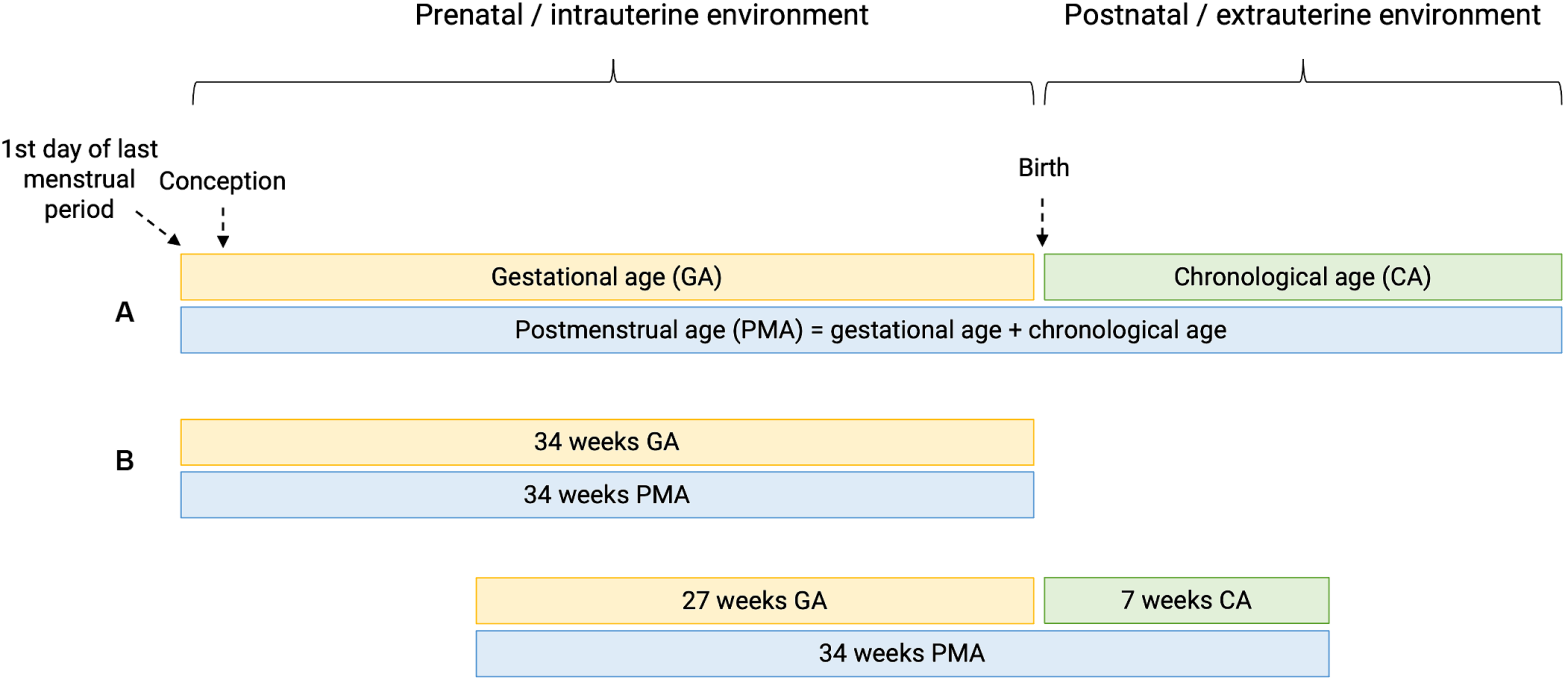
Perinatal definitions. **(A)** Gestational age (GA) is the time elapsed since the first day of the last menstrual period before pregnancy. Chronological age (CA) or postnatal age is the time elapsed after birth (days, weeks, months, years). Postmenstrual age (PMA) is the gestational age plus the chronological age. **(B)** Hypothetical cases of two patients having the same PMA, with different GA and CA.

Preterm birth or prematurity is defined as birth before the gestational age (GA) of 37 weeks. Gestational age (expressed in weeks and days) is calculated from the first day of the last menstrual period before pregnancy (Figure 1). In a regular menstrual cycle of 28 days, this is 14 days before the ovulation/conception date. This method has historically been used because women are frequently unaware of their ovulations but are of their period. In addition, antenatal ultrasound in the first trimester is used to determine the gestational age more accurately, accounting for irregular cycles and patient errors (16). Depending on the GA of the neonate at birth, different preterm age groups are defined (17). Extremely preterm infants, very preterm infants, moderately preterm infants and late preterm infants are born, respectively, at less than 28 weeks, between 28 and 31 6/7 weeks, between 32 and 33 6/7 and between 34 and 36 6/7 weeks GA (Table 1). The time elapsed after birth is called chronological age (CA) (days, weeks, months, years) or postnatal age, and when this is combined with GA, we refer to it as the postmenstrual age (PMA) (weeks). (Figure 1).

**Table 1:** Definitions.

Terminology	Definition	Time of diagnosis
**Prematurity or preterm birth**	Birth before the gestational age (GA) of 37 weeks	Postnatal
Extremely preterm	< 28 weeks GA	
Very preterm	28 weeks – 31 weeks 6 days	
Moderately preterm	32 weeks – 33 weeks 6 days	
Late preterm	34 weeks – 36 weeks 6 days	
**Small for gestational age (SGA)**	Birth weight less than the 10th percentile for that gestational age and gender	Postnatal
**Low birth weight (LBW)**	Absolute birth weight less than 2,500 g	Postnatal
**Intra-uterine growth restriction (IUGR)** (= fetal growth restriction (FGR))	Impaired biometry (estimated fetal weight and/or abdominal circumference below the 10th percentile) or deflecting growth on prenatal ultrasound	Prenatal

Prematurity is the main cause of mortality in children under 5 years of age, predominantly caused by pulmonary-related complications, and preterm infants, in particular extremely preterm infants, can suffer from increased long-term morbidity compared to full-term infants due the immaturity of their organ systems. Common health problems in this population include growth impairment, chronic respiratory disorders (e.g., asthma), vision problems and neurodevelopmental impairment (18).

Preterm birth can occur either spontaneously or through medical induction. Most of the cases are spontaneous due to preterm labor, preterm prelabor rupture of membranes (PPROM) or more rarely cervical insufficiency, but still about 30–35% of preterm deliveries are medically induced. The latter consists of a medical decision taken when continuing the pregnancy is considered too dangerous for the mother and/or fetus, for example in the case of pre-eclampsia (19). While frequently no specific etiology for preterm labor or PPROM can be found, a wide range of risk factors have been identified, including maternal characteristics (low socio-economic status, extremes in maternal ages, low or high Body Mass Index (BMI), previous cervical surgery, tobacco use), obstetrical risk factors (uterine overdistention, uteroplacental insufficiency, previous preterm delivery, assisted reproduction, multiple gestation) but also stress and inflammation (e.g., chorioamnionitis) (17, 19). Recently, a meta-analysis provided evidence for a relation between air pollution and preterm birth (20). Additionally, biological and genetic markers are being assessed in the quest for better understanding the mechanisms triggering prematurity (19).

As this review focuses on the renal consequences of preterm birth, we believe it is important to make the distinction with other entities including small for gestational age (SGA), low birth weight (LBW), and intra-uterine growth restriction (IUGR), since these conditions have been associated with low nephron number (21, 22). SGA is detected postnatally when the birth weight is less than the 10th percentile for that gestational age and gender. This can be due to a pathological growth restriction (i.e., IUGR) or the baby can be constitutionally small due to small parents, congenital malformations or chromosomal abnormalities. IUGR is a prenatal diagnosis detected by ultrasound showing impaired biometry (estimated fetal weight and/or abdominal circumference below the 10th percentile) or deflecting growth. Placental insufficiency is the major cause of IUGR, in which a reduced utero-placental blood flow hampers nutrients and oxygen transfer towards the fetus throughout pregnancy (23). Other causes are maternal illness or undernutrition (24). LBW refers to neonates who are born to small, i.e., with an absolute birth weight of <2,500 g. This ranges from low birth weight (1,500 g – 2,500 g) over very low birth weight (1,000 g – 1,500 g) to extremely low birth weight (500 g – 1,000 g) (25). LBW can be attributed to preterm birth, IUGR or the combination of both and can therefore be considered an umbrella term of these two entities. Note that while LBW, prematurity, IUGR and SGA can overlap, none of them is interchangeable and the use of the correct terminology is critical. In a meta-analysis integrating data on animal and human studies, a clearly increased risk of hypertension in IUGR offspring was observed in rats where clear models of placental insufficiency induced IUGR were used, but this difference was not observed in human studies, where IUGR was often poorly defined using SGA criteria (26).

## 3 Normal course of human kidney development

### 3.1 General overview

The kidney is a derivative of the intermediate mesoderm. Its development starts at approximately week 4 of gestation and it is most commonly completed around 36 weeks (27). During this period, three kidney structures develop from the posterior intermediate mesoderm: the pronephros, the mesonephros and the metanephros or metanephric mesenchyme (MM). The pronephros and the mesonephros are primitive kidney structures that will involute, whereas the metanephros develops into the definitive kidneys. Meanwhile, the mesonephric duct develops from the anterior intermediate mesoderm and elongates downward until it reaches the urogenital sinus, which later forms the bladder. At week 5 of gestation, a mesonephric duct outgrowth known as the ureteric bud (UB) invades and branches into the MM. The cells closest to the branching ureteric tips condense to form a discrete subdomain of the MM: the cap mesenchyme (CM). Over a decade ago, cell lineage analysis in mice using tamoxifen controlled Cre/loxP driven by the progenitor marker genes SIX2, CITED1 and GDNF revealed that the CM represents self-renewing, multipotent nephron progenitor cells (NPC) (28–30). In 2019, the first lineage tracing study within a kidney organoid showed that nephrons within human iPSC-derived kidney organoids are also derived from a SIX2-expressing population (31). This has been further confirmed through pseudo-time trajectory of single cell RNA sequencing data (scRNAseq) of the human fetal kidney and the isolation and *in vitro* characterization of SIX2+/CITED1+ cells from human fetal kidney tissue (32, 33). Interactions between the epithelial UB and mesenchymal NPC result in branching of the UB whereas the NPC population balances between self-renewal and differentiation towards nephron formation. These different cell behaviors (self-renewal/proliferation, maintenance of multipotency, differentiation) of the NPC are coordinated through the action of various signaling pathways (e.g., GDNF, Wnt, FGF, BMP, Notch, Fat, Ras-PI3K and MAPK–ERK) and gene regulatory programs (e.g., SIX1, SIX2, PAX2, OSR1, EYA1, SALL1, WT1 and β-catenin–TCF–LEF) (34, 35). Perturbations to these pathways can lead to premature NPC differentiation and/or loss of the NPC population, although removing specific components of these pathways can lead to different outcomes (36).

To generate a nephron, a group of NPC undergoes mesenchymal-to-epithelial transition (MET) at stereotypical positions to form functionally and morphologically distinct tubular and glomerular epithelial cells. Wnt9b/β-catenin has emerged as the dominant molecular signal initiating this process. The first cellular evidence of progenitor commitment is a clustering of cells into a pretubular aggregate (PTA) beneath the ureteric branch tips. The mesenchymal PTA then transitions to the epithelial renal vesicle (RV). The RV encases a lumen and begins to “unwind” to form the comma-shaped (CSB) and the S-shaped bodies (SSB), ultimately vascularizing at the proximal end into a capillary loop and finally the mature nephron. The neonephron fuses with the ureteric tip shortly after the MET event to generate a continuous luminal interconnection between the two epithelial networks, resulting in the functional nephron. This process is repeated around 1 million times during human kidney development. Whereas mouse nephrogenesis is rapid and the progression from nephron progenitor to S-shaped body is approximately 24 h, the equivalent human development is estimated to be 3–8 times slower (37). This provides temporal resolution to the human nephrogenesis process. In fact, we now know that an order exists by which NPC differentiate and that this order corresponds to the positioning of the cells along the distal-to-proximal axis of the nephron. Cells that are added first generate the most distal segments of the nephron; cells that are added thereafter are incorporated in a distal-to-proximal direction. This model, known as the gradual recruitment model, implies that podocytes and the parietal epithelium are the last recruits into the forming nephrons.

Through the advent of (spatial) single-cell modalities such as transcriptomics, epigenetics, metabolomics, it has now been reported that the adult kidney contains up to 41 renal and 32 non-renal populations (33). As we are entering into a new era of cell type definition that goes beyond microscopical morphological appearance, more cell types are expected to be discovered along with the molecular signatures and lineage pathways driving the development of these cells.

### 3.2 The three phases of nephrogenesis

Overall, nephrogenesis proceeds in three phases. In the first phase, NPC form nephrons through a process associated with UB bifurcation, known as branching morphogenesis. Each branch generates between two and three nephrons. In mice, branching morphogenesis persists until approximately postnatal day 2 and generates around 14.000 nephrons. Shortly thereafter, the remaining NPC pool in each UB tip-associated niche differentiates *en masse* into nephrons, thereby completing nephrogenesis in a total period of 10 days. Branching nephrogenesis produces ~33.000–42.000 nephrons in humans and is followed by post-branching nephrogenesis (38). The first post-branching phase is known as arcading, observed between 15 and 22 weeks of gestation in humans (38, 39). Arcades are morphologically characterized by immature nephrons directly connected to each other in chains that drain into a single collecting tubule. During arcading, the UB does not branch, nor does it elongate significantly. The second post-branching phase is known as lateral branch nephrogenesis that occurs from 22 weeks of gestation onwards. Lateral branch nephrogenesis is typified by an elongating UB stalk with an active UB tip that induces nephrons periodically, each directly connecting to the ureteric stalk as it undergoes MET. Lateral branch nephrogenesis, which occurs during the third trimester of pregnancy, is the most active period of fetal nephrogenesis in humans (and other primates), during which more than 60% of nephrons are formed. It is this phase most affected by prematurity. Post-branching nephrogenesis in the human kidney creates 15–75 nephrons at or near each of the 33.000–42.000 terminal branch tips. As in rodents, human nephrogenesis ends with the coordinated differentiation of all remaining NPC in multiple independent niches. Consequently, the population of nephrons generated during the developmental period is the population for life.

### 3.3 Cessation of nephrogenesis

During kidney organogenesis the number of NPC surrounding each ureteric tip gradually declines, preceding a final wave of synchronous differentiation followed by cessation of nephron formation (40). Through the assessment of kidney tissue from deceased fetuses, it was shown that this timing of cessation of nephrogenesis in the human kidney is variable, ranging from as early as 32 weeks of gestation to term gestation (37 weeks or later) (27, 41, 42). In these studies, nephrogenesis was considered ongoing if there was morphological evidence of a condensed mesenchyme and/or immature nephron structures (RV, CSB, SSB) in the outer renal cortex. This is often referred to as the “nephrogenic zone”.

**Figure 2:**
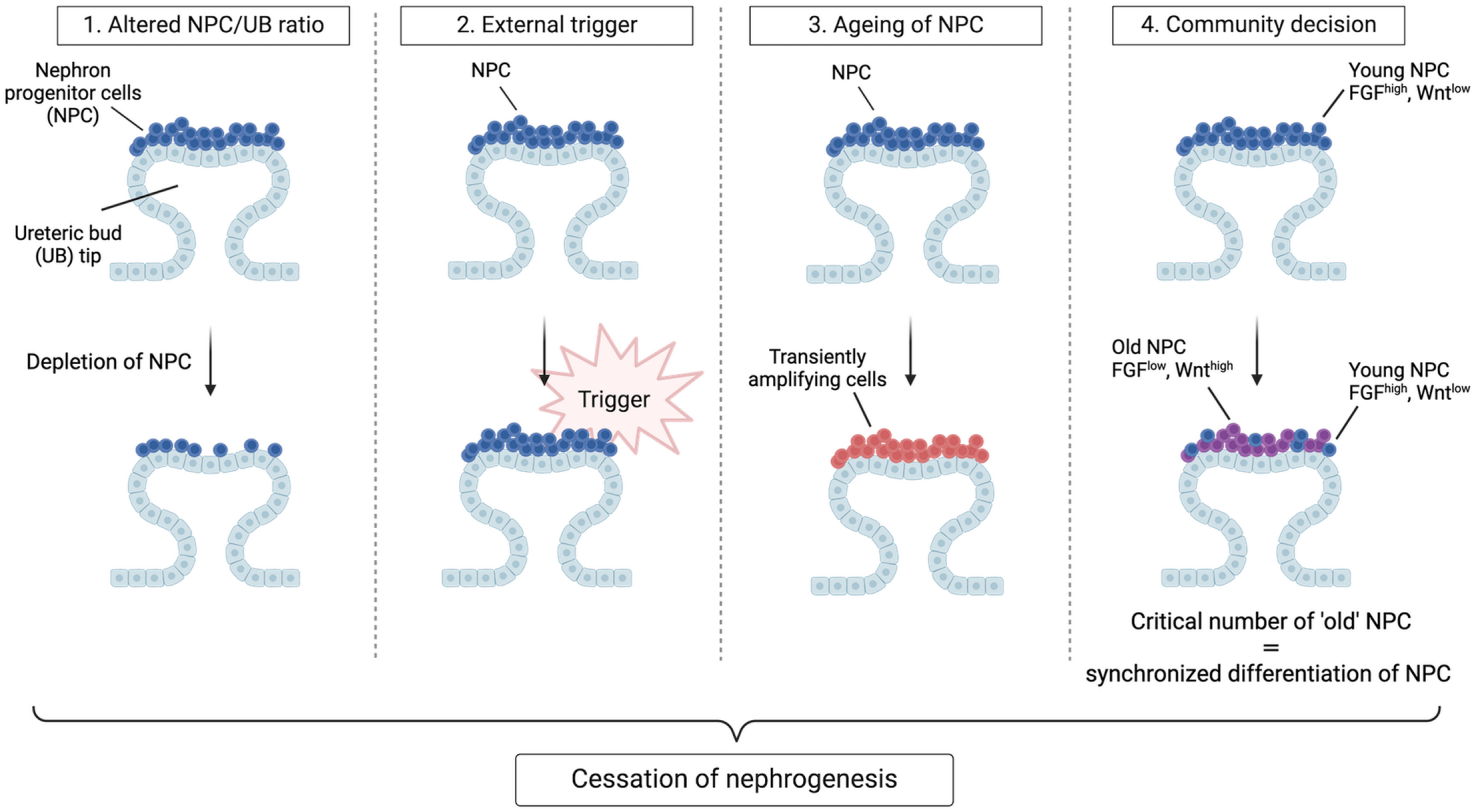
Hypotheses for cessation of nephrogenesis. Four models have been envisaged to explain synchronous cessation of nephrogenesis. In the first model, nephrogenesis terminates when a certain ratio of NPC to UB tips is achieved through gradual recruitment and hence depletion of NPC. In this model, a change in the concentration of critical niche factors brought about by the reduction in CM/UB ratio ends nephrogenesis by shifting the balance towards differentiation. In the second model, a *de novo* active trigger abruptly ends self-renewal of the homogenous progenitor population and causes an exit from the progenitor-like state. In the third model, the NPC population gradually transitions to more committed, transiently amplifying cells, causing a depletion of the self-renewing fraction within the cap mesenchyme to the point that no true progenitors remain. In the last model, cessation of nephrogenesis is a community decision taken once a critical number of NPC have acquired an “old” phenotype. In this model, “young” NPC have high transcript levels of niche-retention factors (e.g., FGF9 and FGF20) whereas “old” NPC have an increased transcription and translation of Wnt agonists, resulting in a stronger perception of the Wnt signals arising from the ureteric bud, promoting NPC differentiation and niche exit. Once a critical number of NPC have exited the niche, all the remaining cells follow.

The apparent synchronization of nephrogenesis cessation across all niches has prompted a search for the trigger(s) leading to this event. Three hypotheses have initially been proposed. The first is an altered NPC/UB ratio; the second is an active, external trigger that ends NPC self-renewal; the third is the termination of nephrogenesis through a gradual depletion of the self-renewing NPC to the point that no “true progenitors” remain. In studies that randomly eliminated 40% of NPC via diphtheria toxin A-induced apoptosis in mice, the number of nephrons was lowered and branching was delayed but cessation timing was not altered (30, 43). These findings demonstrate that the cessation mechanism is independent of the ratio of NPC to UB tips, the size of the NPC population and the number of branching events. Regarding the other two hypotheses: if we consider NPC to resemble conventional, homogenous stem cells, a loss of extrinsic, permissive niche factors could promote exit from the NPC niche. Alternatively, if we consider NPC to resemble transiently amplifying cells that age, an internal “clock” mechanism may control the timing of NPC (44). These hypotheses were evaluated by Kopan and colleagues who transplanted “young” and “old” mice NPC (i.e., NPC isolated from kidneys at different embryonic ages) into young NPC niches (45). The results of their study were inconsistent with either hypothesis: although the ability of NPC to engraft worsened with increasing age, engraftment of “old” NPC was improved if they were surrounded by “younger”, FGF20-producing NPC. Moreover, they observed progressive age-dependent changes in older populations of NPC, including decreased FGF9/20 and elevated mTOR signaling. Another study by the same group demonstrated that NPC modify their responsiveness to Wnt signals over time by increased translation and stability of Wnt/Fzd complexes in older niches, eventually reaching a tipping point where self-renewal processes are outweighed by signals that drive synchronized differentiation (46). Thus, rather than a cell-intrinsic or cell-extrinsic mechanism, these results suggest the presence of a community effect-based mechanism that controls the timing of nephrogenesis cessation: once a critical number of cells exit the niche, all the remaining cells follow. This mechanism is referred to as the “tipping point” model and provides a fourth concept for the cessation of nephrogenesis (Figure 2). Nevertheless, these results do not rule out possible roles of extrinsic triggers, such as birth, which could work in parallel to or even trigger intrinsic changes in regulating progenitor lifespan.

### 3.4 Nephron endowment

#### 3.4.1 Methodologies for nephron counting

Nephron endowment is defined as the number of nephrons that an individual has at birth. This number constitutes a measure of the overall success of nephrogenesis and does not change until loss occurs with ageing or disease. To estimate or quantify nephron numbers, different techniques exist. The current gold standard is the physical dissector/fractionator method by Sterio, a method that involves sectioning a kidney into progressively smaller pieces, then counting glomeruli by systematic random sampling. This approach requires no knowledge or assumptions of glomerular geometry (size, shape), and when used correctly, provides unbiased, accurate estimates. However, this technique is time-consuming, laborious and requires access to the entire kidney at autopsy. Another histological method, although less accurate, is the acid maceration method which uses acid to digest the entire kidney; a portion of the disassociated glomeruli are then counted. This method also relies on postmortem analysis after destruction of the kidney. Both the physical dissector/fractionator method and the acid maceration method do not allow for (longitudinal) analysis in living patients, and do not easily allow 3D visualization and integration of the renal microstructure. Moreover, in each of these methods nephron numbers can only be estimated because only glomeruli in a small portion of the kidney are counted; mathematical equations are used to estimate the total nephron number. Additionally, all visualized glomeruli—regardless of whether they are functional or obsolescent—are counted. To overcome these limitations, MRI-based techniques have been proposed for the non-invasive measurement of nephron number and glomerular size distribution in living patients and are currently under development (47–49). Finally, surrogate measurements of total nephron number have been used to quantify nephrons in living patients including radiological measurement of kidney size, counting of 2D glomerular cross-sections in histological sections, and the medullary Ray Glomerular Counting (RGC). The latter technique consists of counting the number of layers (“generations”) of glomeruli in a line alongside a medullary ray (a bundle of collecting tubules) extending from the inner cortex (earliest formed nephron in the arcade) to the outer cortex (most recently formed individually connected nephron). All of these surrogate measurements lack accuracy to determine actual nephron number.

#### 3.4.2 Nephron number in humans

Based on postmortem studies counting glomeruli, it appears that humans on average possess approximately 900.000 nephrons per kidney (50). However, there is substantial inter-individual variation with nephron numbers varying up to 10-fold in healthy subjects. The largest cohort to date was described by Bertram et al. in 2003 and included 398 subjects with a variety of ethnic and racial backgrounds. The average number of glomeruli per kidney reported was 895.711 with a variation between subjects ranging from 210.332 to 2.702.079. In this study, female sex, older age, race (lowest in the Australian Aborigines), and LBW were associated with lower nephron number (51). Analyses of autopsied kidneys from younger patients (< 3 months of age) demonstrated that this variability in nephron numbers is present at birth and not merely a consequence of age-related nephron loss (52). The mechanisms underlying this large variation in nephron endowment are currently unknown. It is likely that (epi) genetic factors, nutritional factors, nephrotoxic exposure, environmental factors (maternal smoking, alcohol), systemic and placental conditions are all contributing factors (44). Notably, the reliance on postmortem samples to estimate most accurately nephron number makes it impossible to determine the normal nephron number among healthy subjects in a completely unbiased manner. In all of the studies reporting nephron numbers in autopsied kidneys, subjects with lower nephron number might have had unrecognized CKD.

## 4 Preterm birth and kidney development: observations and underlying pathogenesis

Nephron number is strongly correlated with gestational age and, as previously mentioned, the majority (*circa* 60%) of nephrons form exponentially in the third trimester of pregnancy (27). Therefore, at the time preterm infants are born, kidney development may still be fully ongoing. Upon delivery, preterm infants are suddenly exposed to an unfamiliar external environment that is likely suboptimal for ongoing organ development. Moreover, although still structurally and functionally immature, the newborn’s kidneys must also function independently for the first time. Considering all these factors, one might expect that the normal trajectory of kidney development is disrupted in premature infants.

There is now considerable evidence that nephrogenesis can continue postnatally although in an aberrant way. Sutherland et al. performed an autopsy study in 28 preterm neonates (GA at birth ranging from 24 to 35 weeks, postnatal age ranging from 2 to 68 days) showing the presence of a nephrogenic zone with developing glomeruli in kidney tissue after birth (42). However, compared to PMA-matched stillborns, preterm infants exhibited accelerated postnatal renal maturation with a reduced nephrogenic zone width and a reduced percentage of immature nephron structures. The number of glomerular generations was significantly increased compared to the controls, but preterm kidneys exhibited an enlarged renal corpuscle cross-sectional area and around 13% of glomeruli appeared to have an abnormal morphology (i.e., dilated Bowman’s space, shrunken glomerular tuft). Abnormal glomeruli were only present in the outer cortex, suggesting that it is the newer glomeruli formed in the extra-uterine environment that are unlikely to be fully functional. These structural changes have been observed in animals as well and are possibly caused by compensatory glomerular hypertrophy to cope with the postnatal functional demands (53). This study did not report on the latest postnatal age at which nephrogenesis could still be observed in preterm infants. Contrary to this study, autopsy studies by Rodriguez et al. and Faa et al. found that compared with term-born infants, the number of glomerular generations, and hence nephron endowment, was reduced in preterm infants (54, 55). Rodriguez et al. found that RGC were significantly decreased in all preterm infants as compared to term controls and correlated with gestational age. It is important to note, however, that a large proportion of the neonates in the preterm group had IUGR, which is a well-known cause of low nephron endowment (21, 22). In a non-human primate model of preterm birth in the absence of IUGR, it was observed that nephron endowment was not affected by preterm birth. In the study by Rodriguez et al. there was histological evidence of active glomerulogenesis up until 39 days postnatal age.

Very recently, Carpenter et al. characterized postnatal nephrogenesis in an autopsy study of 7 preterm infants without IUGR (mean GA at birth 25 weeks) older than 40 days CA and compared it to 8 PMA-matched controls (mean GA at birth 33 weeks). Importantly, they investigated the duration of nephrogenesis both histologically and at the molecular level using SIX1+/SIX2+/RET+ immunostainings. Unlike SIX1 expression, which is maintained throughout nephrogenesis both in the NPC as well as in the nascent nephron (56), minimal SIX2 staining was seen in these preterm samples. Therefore, they relied on RET+ signal as the most accurate surrogate for active nephrogenesis. They demonstrated that although the timing of cessation of nephrogenesis was still 2 weeks earlier than in the PMA-matched controls, it persisted beyond 40 chronological days and therefore remained within the “normal” window of 32 to 36 weeks PMA. In one infant, there was molecular and histological evidence of postnatal nephrogenesis at 62 days CA. There were no significant differences in the number of glomerular generations between the two groups but preterm infants had larger kidneys, larger glomeruli and significant proximal and distal tubule hypertrophy. Different to what had been observed previously, it appeared that the more mature and deep glomeruli at the medullary border, formed antenatally, were even more stressed than cortical, postnatally formed nephrons. Overall, these data suggest a window of both vulnerability and potential protection beyond the first 40 days of life.

Many players surrounding preterm birth could contribute to the disturbances in nephrogenesis described above. Unfortunately, the preclinical data is scarce. Conceptually, these factors could be classified as prenatal, perinatal and postnatal insults. Prenatally, preterm infants might have been exposed to factors that precipitated preterm delivery such as placental insufficiency, gestational diabetes, preeclampsia but also inflammatory conditions. Chorioamnionitis, a bacterial intrauterine infection affecting fetal and maternal tissues, is the single most common cause of preterm delivery, with reported rates up to 50% (57). Hypothetically, the inflammatory response induced by such a bacterial insult may impede nephrogenesis through the disruption of the VEGF and renin-angiotensin-aldosterone system (RAAS) signaling pathways, the two major pathways for glomerular and peritubular vasculogenesis (58, 59). Moreover, pro-inflammatory signals cytokines may lead to renal fibrosis, further causing a reduction in the number of functional glomeruli. Perinatal administration of nephrotoxic medications and/or medication that promote organ maturation could also contribute to impaired kidney development. For example, exposure to antenatal glucocorticoids, which are commonly administered before preterm delivery to aid postnatal respiratory function, is associated with renal functional maturation and reduced nephron endowment in clinical studies and animal models of prematurity (53, 60–62). Moreover, substantial hemodynamic changes occur shortly after birth with typically an increase in systemic and renal blood pressure and a reduction in renal vascular resistance (63). This contrasts with the intra-uterine environment which is characterized by low oxygen tension and low renal blood flow and pressure. Postnatally, preterm infants frequently have to face up a stressful extra-uterine environment that involves administration of parental nutrition, supplemental oxygen exposure, mechanical ventilation, nephrotoxic medications (e.g., aminoglycoside for bacterial infections, NSAID for closure of patent ductus arteriosus) but also episodes of acute kidney injury (AKI). These factors could all be unfavorable for the developing, “immature” kidney.

**Figure 3:**
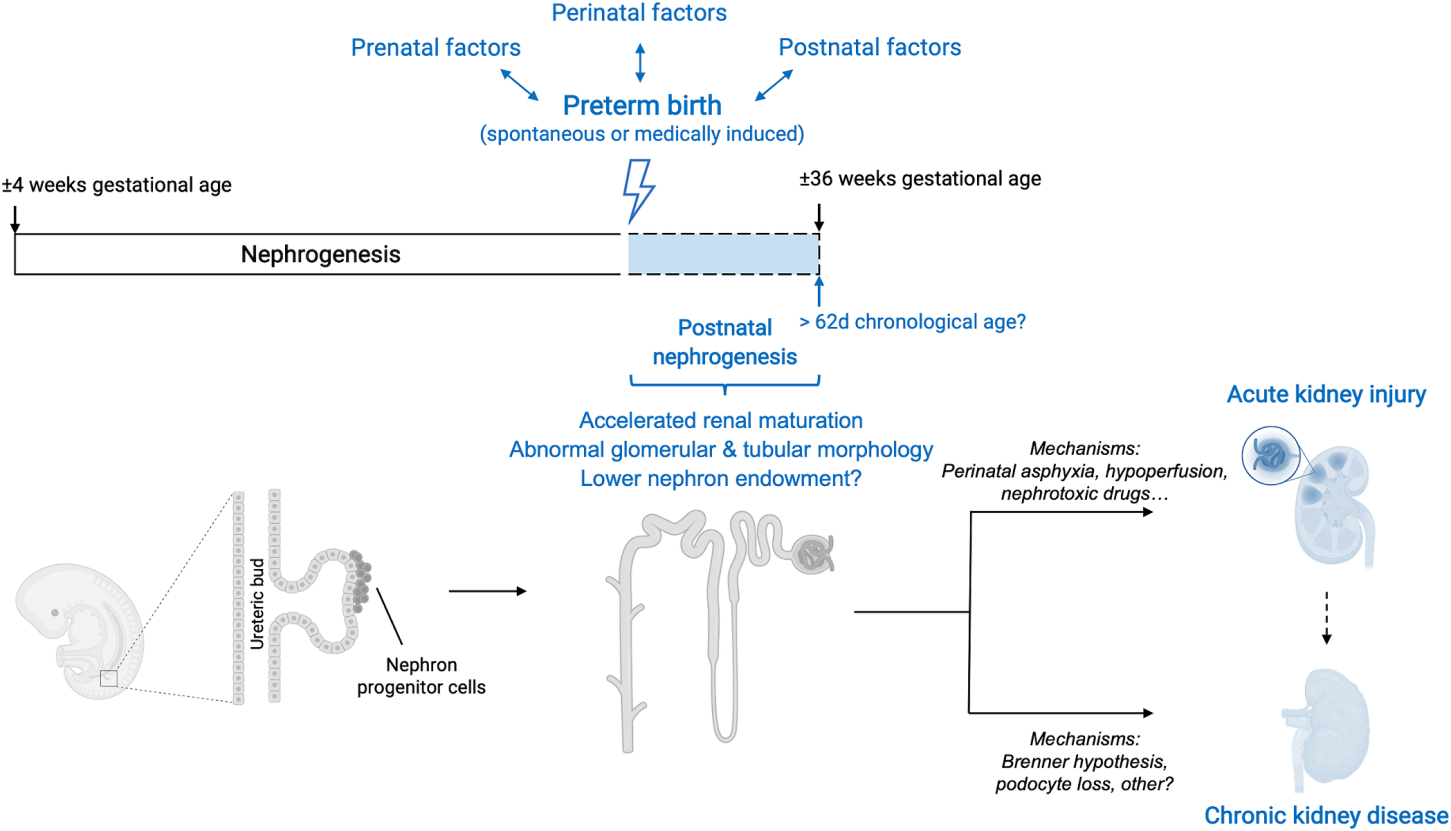
Overview of interactions between preterm birth, nephrogenesis and kidney disease. Preterm birth, along with its associated prenatal, perinatal and postnatal factors, causes a disruption in the normal trajectory of human kidney development. Although after preterm birth nephrogenesis can persist postnatally, the course is accelerated, and glomerular generation and tubular maturation are impaired. Overall, these histological abnormalities translate into a lower number of functional nephrons. The effect of preterm birth on absolute nephron number is currently still unclear. The clinical renal consequences of preterm birth are both short-term and long-term and include an increased risk of neonatal acute kidney injury (AKI) as well as a higher risk of developing chronic kidney disease (CKD) later in life. The mechanisms underlying these clinical manifestations are likely multifaceted and an area of active research. Additionally, the occurrence of neonatal AKI is, by itself, a risk factor for developing CKD later in life and therefore acts as a positive effect modifier in the relationship between prematurity and CKD.

In conclusion, the evidence suggests that kidney development can persist postnatally after preterm birth for as long as 62 chronological days but does not follow the normal growth trajectory of that *in utero*. Nephrogenesis is accelerated and a significant proportion of glomeruli have an abnormal renal morphology, leading to an overall reduction in the number of functional nephrons. Therefore, kidneys of humans born prematurely do not reach the full nephrogenic potential they would achieve if born at term. The effect of preterm birth on nephron number is currently unclear. In addition to impaired glomerulogenesis, tubular maturation can also be disrupted, but to this date little research has been performed on this matter (Figure 3).

## 5 Preterm birth and consequences on kidney health: the clinical evidence

Fewer layers of larger glomeruli with more histologic abnormalities suggest that premature infants have a reduced number of functional nephrons early on in life. Luyckx and Brenner proposed that this lower amount of nephrons leads to an increase in glomerular size due to hypertrophy, consistent with hyperfiltration in the remaining nephrons (64). Glomerular hyperfiltration is driven by changes in hemodynamics and intraglomerular pressure leading to an increase in single nephron glomerular filtration rate (GFR) (65). This maladaptive response further causes progressive kidney injury through the activation of the RAAS system, occurrence of hypertension and the direct effect of hyperfiltration-related mechanical forces, creating a vicious cycle. Hyperfiltration manifests clinically as microalbuminuria and accelerated loss of renal function, and histopathologically as glomerulosclerosis and tubulointerstitial inflammation. A kidney with reduced nephron number has also less functional reserve to adapt to dietary excesses or to compensate for renal insults.

Various epidemiological studies have reported that LBW, which encompasses prematurity and IUGR, is associated with an increased risk of kidney-related outcomes (hypertension, albuminuria, reduced estimated GFR (eGFR)) in childhood and adulthood (66–77). A systematic review and meta-analysis including 18 observational studies with data from more than 2 million individuals demonstrated the statistically significant association between LBW and a higher risk for CKD, end-stage renal disease (ESRD), low eGFR and albuminuria (78). Although the GA was not systematically investigated in most of these studies, preterm birth accounts for approximately 80% of LBW newborns and thus contributes to a significant portion of these patient cohorts (79, 80). Only more recently the contribution of IUGR and preterm birth to the development of kidney complications has been investigated as separate entities. Importantly, this distinction is essential for the identification of who is exactly at risk. In this section, we review the literature supporting the association between preterm birth and decreased kidney function on short-and long-term.

### 5.1 Preterm birth and neonatal acute kidney injury

There has been a considerable amount of work highlighting the higher incidence and consequences of AKI in preterm neonates and neonates with LBW (81–86). A systematic review and meta-analysis by Wu et al. including fifty studies and a total of 10.744 patients with LBW and/or prematurity revealed that the overall prevalence of AKI among these neonates is 25% (95% CI 20–30%), although there was a great variation between studies with reported AKI prevalence ranging from 7 to 68% (87). Subgroup analysis showed that the rate of AKI among patients with GA lower than 32 weeks was 26% (95% CI = 18–34%). Importantly, mortality rates of neonates with AKI were significantly higher than those without AKI (OR = 7.13; 95% CI 5.91–8.60; *p* < 0.01). The largest study included in this meta-analysis reported the prevalence and severity of AKI in relation to gestational age and birth weight in 923 extremely low gestational age neonates (ELGAN, < 28 weeks GA) (88). They compared the prevalence and severity of AKI by specific time frames (first, second, and after the second postnatal week) across four GA categories (24, 25, 26, and 27 weeks) and found that approximately 38% (95% CI = 34.8–41.3%) of the cohort developed at least one episode of stage 1 or higher AKI and that 18.2% (95% CI = 15.7–20.7%) had at least one episode of stage 2 or higher AKI (Table 2). Moreover, 24 weeks GA neonates had two times and three times the rates of severe AKI compared to 26 and 27 weeks GA infants, respectively.

**Table 2:** Neonatal AKI KDIGO classification.

AKI stage	Serum creatinine (SCr) criteria	Urine output criteria
No AKI	No change in SCr or rise <0.3 mg/dL	
1	SCr rise ≥0.3 mg/dL rise within 48 h or SCr rise ≥1.5–1.9 × baseline SCr* within 7 days	<0.5 mL/kg/h for 6–12 h
2	SCr rise ≥2.0–2.9 × baseline SCr*	<0.5 mL/kg/h for >12 h
3	SCr rise ≥3 × baseline SCr* or SCr ≥ 2.5 mg/dL or kidney support therapy utilization	<0.3 mL/kg/h for ≥24 h or anuria for ≥12 h

^*^ Baseline SCr defined as lowest previous SCr value.

Whether the higher mortality observed in neonates with AKI is a direct consequence of AKI or merely a reflection of the severity of illness of these infants was investigated in a large prospective multicenter cohort study including 900 ELGAN (86). In this cohort, 19% of ELGAN had at least one episode of severe AKI (i.e., stage 2 or 3 AKI) and 7% had stage 3 AKI. Those with stage 3 AKI had an almost four-fold increased risk of death (HR 3.88; 95% CI = 1.26–11.96). This association was independent of multiple important confounders including GA, sex, maternal, race, ethnicity, 5 min Apgar, need for chest compressions, intubation, epinephrine use, necrotizing enterocolitis, intraventricular hemorrhage and sepsis. The associations between severe AKI and mortality were strongest when AKI occurred after the first 2 weeks of life, compared with earlier time points. In total, 50% of the deaths occurred after day 14, which supports the importance of ongoing monitoring of kidney function beyond the first few weeks of life.

In addition to increased mortality, neonatal AKI also places the infant at a higher risk of CKD later in life due to maladaptive repair processes. Observational longitudinal studies have shown relatively high rates of CKD following neonatal AKI with at least 20% of patients developing a GFR of less than 60 mL/min/1.73 m^2^ upon follow-up (89). In the studies that did not identify any patients with a GFR of <60 mL/min/1.73 m^2^, a mildly reduced GFR (60–90 mL/min/1.73 m^2^) was reported in an additional 5–40% of the study populations. In studies that measured urinary protein excretion, proteinuria was identified in 12–66% of these patients.

The great variation in AKI prevalence as observed in the meta-analysis by Wu et al. is likely due to differing AKI definitions, the postnatal day chosen for baseline serum creatinine, and variation in the number and timing of serum creatinine measurements. Indeed, across these studies varying definitions of AKI were used including the Kidney Disease Improving Global Outcomes (KDIGO) definition, the Acute Kidney Injury Network (AKIN) definition, the pediatric risk injury failure, loss of kidney function (pRIFLE) definition, a combination of these or other definitions of AKI. To date, the neonatal modified KDIGO definition remains the consensus definition that should be utilized in research and clinically to diagnose and stage AKI (Table 2) (90, 91). On the other hand, the selection of the baseline, steady-state serum creatinine remains a topic of discrepancy. Indeed, serum creatinine levels are highly dynamic in the early postnatal period, which complicates the use of a “static” definition for neonatal AKI (92). Serum creatinine is high in the first days of life and are reflective of a naturally low GFR (< 35 mL/min per 1.73 m^2^) and transfer of maternal creatinine. In more premature infants, the serum creatinine level often rises above maternal creatinine levels, potentially due to tubular reabsorption of creatinine in the context of immature tubular function (89, 93). Serum creatinine then declines at varying rates over the first few weeks of life, with the slope of this decline directly related to the GA of the infant. The role of potential AKI occurring in the context of this early, transient rise of serum creatinine in premature infants is not known. Some have advocated for a more dynamic evaluation of neonatal kidney function, looking at the rate of serum creatinine decline in combination with serum creatinine thresholds (94).

### 5.2 Preterm birth and chronic kidney disease

Clinical signs of kidney disease among patients born prematurely have been detected in patients’ cohorts of different age categories, spanning from the early postnatal period to adulthood (Table 3). Schreuder and colleagues retrospectively analyzed amikacin clearance on the first day of life as an estimation of GFR in 161 neonates born prematurely. They found that a lower birth weight and a lower GA correlated with a lower clearance of amikacin, after correction for other factors (95). Allegaert et al. investigated amikacin and vancomycin clearance, two drugs which are almost exclusively eliminated by renal clearance, in preterm neonates born SGA or appropriate for gestational age (AGA). Renal drug clearance was significantly lower in preterm neonates born SGA than in AGA controls. This reduced clearance was observed not only at birth but also up to the postnatal age of 4 weeks (102). In this study, no comparison was made with clearance in term neonates. Another study found in a large prospective cohort of 565 infants born at extremely low GA (< 28 weeks) that 18% had CKD (eGFR <90 mL/min/1.73 m^2^), 36% had albuminuria, 22% had an elevated systolic blood pressure, and 44% had an elevated diastolic blood pressure at 2 years of age (85). The prevalence of CKD was lower with increasing GA with the highest prevalence (i.e., 26%) in infants born at 24 weeks GA. Kwinta et al. followed 78 infants with a median GA of 27 weeks and 38 full-term age-matched controls up to a median age of 6.7 years. The authors reported that cases had significantly higher serum cystatin C levels and smaller kidneys, as assessed by ultrasonography, than the controls, suggesting that kidney growth may be stunted after preterm birth (97). Similarly, Raaijmakers and colleagues found that compared to healthy controls born at term, prematurely born children (GA 23–33 weeks) who had an extremely low birth weight (ELBW, < 1,000 g) had higher blood pressure, smaller kidneys and lower eGFR at a mean age of 11 years (99). Interestingly, this higher blood pressure was associated with lower plasma renin activity, suggesting that the pathogenesis of hypertension after premature birth is unlikely to be mediated through a mechanism dependent on the renin-aldosterone-angiotensin system, in contrary to what has previously been proposed.

**Table 3:** Observational studies reporting on the association between prematurity and chronic kidney disease.

Study	Study type	Country	Participants & sample size	Age of participants	Main findings
Keijzer-Veen (73)	Cohort study	The Netherlands	422 19-year-old subjects born very preterm (< 32 weeks GA)	19 years	In young adults born very preterm with IUGR, there was a negative association between the extent of IUGR and renal function: subjects born with IUGR, had lower GFR, higher serum creatinine concentration and higher microalbumin excretion at the age of 19 years. No convincing relation was found between GA and renal function.
Schreuder (95)	Cross-sectional study	The Netherlands	161 neonates treated with amikacin (mean GA 32 weeks)	1 day	Birth weight and gestational age are correlated with the clearance of amikacin, after correction for other factors.
Keijzer-Veen (96)	Cross-sectional study	The Netherlands	51 20-year-old subjects born very preterm (23 SGA, 29 AGA); 30 full-term controls	20 years	20-year-old female individuals born very prematurely (both SGA and AGA) had significantly smaller renal length and volume, compared with age-matched controls. IUGR had only a small, non significant effect on renal size.
Kwinta et al. (97)	Cross-sectional study	Poland	78 ELBW infants with median GA 27 weeks; 38 full-term controls	6–7 years	ELBW infants had significantly higher serum cystatin C and lower mean renal volume.
Hirano (98)	Case–control study	Japan	381 pediatric CKD cases (81 born <37 weeks GA); 20.619.622 controls	3 months to 15 years	Both birth weight and gestational age were strongly associated with childhood-onset CKD. RR for pediatric CKD was significantly higher in th LBW group (RR: 4.10; 95% CI 3.62–5.01); 82 patients with pediatric CKD were born, and as with LBW, a strong correlation was observed between prematurity and CKD (RR: 4.73; 95% CI 3.91–5.73).
Raaijmakers (99)	Cohort study	Belgium	93 subjects born preterm (GA = 23 to 33w) with a birth weight of <1,000 g; 87 controls born at term	11 years	Subjects born preterm had significantly lower renal length and GFR and higher systolic and diastolic blood pressure. Plasma renin activity (PRA) was significantly lower in subjects born preterm compared to controls.
South (100)	Cross-sectional study	USA	96 14-year-olds born preterm with very low birth weight compared to 43 term	14 years	Preterm birth was significantly associated with higher blood pressure and lower eGFR compared to term controls. Overweight/obesity and sex modified the strength of these associations.
Eriksson et al. (68)	Cohort study	Finland (Helsinki)	20.431 participants followed up from birth	Death or 86 years	Small body size at birth is associated with increased risk for developing CKD in men. Prematurity was also associated with an increased risk for CKD in women.
Crump et al. (67)	Cohort study	Sweden	4.186.615 singleton live births	43 years	Preterm birth and extremely preterm birth (< 28 weeks GA) were associated with nearly twofold and threefold risks of CKD, respectively. An increased risk was observed even among those born at early term. The association between preterm birth and CKD was strongest at ages 0–9 years, then weakened but remained increased at ages 10–43 years. These associations affected both males and females and did not seem to be related to shared genetic or environmental factors in families.
Gjerde et al. (101)	Cohort study	Norway	2.679.967 singleton live births	50 years	Only subjects with at least two or three of the risk factors LBW, SGA or preterm birth have increased risk for ESRD.
Hingorani et al. (85)	Cohort study	United States	565 extremely low gestational age neonates (ELGANs) (born <28 weeks GA)	2 years	Gestational age, birth weight z-score, and prenatal steroids were significantly associated with an eGFR <90 mL/min per 1.73 m^2^.

In the adult population, a large Swedish prospective cohort study that included 4.186.615 live births found that the risk of CKD was 2-to 3-fold higher among young adults (up to age 43 years) who had been born preterm compared to those born at term, and CKD risk correlated inversely with gestational age (67). Despite an ongoing statistically significantly higher risk for CKD in all age groups, the association between CKD risk and prematurity was the strongest at ages 0–9 years (HR 7.29, 95% CI = 4.26–12.47, *p* < 0.001), then weakened but remained increased at ages 10–19 years (HR 1.97, 95% CI = 1.57–2.49, *p* < 0.001) and ages 20–43 years (HR 1.34, 95% CI 1.15–1.57, *p* < 0.001). In this study, preterm birth was an independent risk factor for CKD, but the risk of CKD was highest among participants who were born prematurely and had IUGR. They also found a significantly increased risk of CKD among those born at early term (HR 1.30, 95% CI 1.2–1.4). Importantly, based on co-sibling analyses, these findings did not seem to be due to shared genetic or environmental factors in families. Similarly, the Helsinki birth cohort that included 20,431 participants who were followed from birth to either death or age 86 years demonstrated that CKD at a median age of 65 years was significantly associated with birth before 34 weeks of gestation (HR 2.6, 95% CI = 1.3–4.6), especially among female infants (HR 3.2, 95% CI = 1.4–7.4) (68). In a Norwegian study including 2.679.967 subjects aged 18–50 years old, the risk of ESRD was only increased in individuals who had at least 2 of the 3 risk factors LBW, SGA or preterm birth. This underlines the importance of making the distinction between different subcategories instead of using LBW as an umbrella term. Lastly, in addition to the increased predisposition to CKD, prematurity also associates strongly with hypertension, impaired glucose homeostasis/diabetes and dyslipidemia, which are all major risk factors for the development of CKD (58).

## 6 Preterm birth and consequences on kidney health: animal models and mechanistic insights

The observational epidemiological studies described above can only show an association between prematurity and kidney disease. Moreover, examining the impact of prematurity alone is challenging as many infants born prematurely also have IUGR as well as multiple medical problems and exposure to nephrotoxins. Even in prospective cohort studies that minimize these confounders, a strong association between prematurity and kidney disease does not prove causation. Moreover, clinical mechanistic evidence on the effect of preterm birth on the kidneys is limited due to the inability to longitudinally assess the kidney tissue in a non-invasive way in patients and the lack of reliable and standardized methods to detect the progression, disruption, or cessation of nephrogenesis. This highlights the importance of animal studies. However, although the Brenner hypothesis was proposed decades ago, few studies have investigated the mechanistic link between preterm birth and kidney development and disease.

Baboons in many ways are an ideal model to study the effect of preterm birth (103). Neonatal baboons can be delivered and resuscitated in a manner similar to humans. However, the cost and resources necessary for the success of this model limit its widespread use and prolonged follow-up of these animals. Research with the baboon model of prematurity showed that the number of glomerular generations was not affected by preterm birth but presented significant decrease in glomerular density (number of glomeruli per gram of kidney). Moreover, kidneys were significantly enlarged in preterm baboons in comparison with gestational controls. Abnormal glomeruli, with a cystic Bowman’s space and shrunken glomerular tuft, were often present in the superficial renal cortex of both the steroid-exposed and unexposed preterm kidneys (53).

Contrary to research in primates, rodent models can be studied in larger numbers and are relatively inexpensive. Nevertheless, their kidney development is different from humans with nephrogenesis continuing for 5 to 7 days postnatally. Yet, it has been shown in a mice model that premature birth alone still has a profound effect on nephrogenesis; similarly to what has been observed in humans, mice born prematurely exhibit premature differentiation of nephron progenitor cells, a shorter duration of nephrogenesis and lower glomerular density (104). Moreover, in line with the hypothesis that prematurity leads to reduced nephron endowment and correlated hypertension, mice born 1 to 2 days prematurely develop a CKD phenotype by the time they are 5 weeks old with hypertension and albuminuria, and a reduced glomerular number (105).

In addition to the hypothesis that low (functional) nephron number could increase the risk of developing long-term CKD, it seems that podocyte depletion is also involved in this process (106, 107). Preterm birth accelerates podocyte depletion during growth and in turn contributes to a high risk of future CKD development (108). This hypothesis is substantiated by the fact that preterm infants excrete 5-fold more podocytes in urine than corrected GA-matched full-term infants (107). In line, using a rat model of preterm birth, it was found that prematurity leads to a decreased number of differentiated podocytes and accelerated podocyte differentiation. Also, the number of other types of inherent kidney cells was decreased in this preterm birth model. Another study performed in mice found that preterm birth was associated with fewer podocytes per glomerulus and a disorganized pattern of WT1+ cells in the immature bodies in the nephrogenic zone, suggesting a podocytopathy may underlie the transition from preterm birth to CKD (104).

The molecular mechanisms underlying the observed accelerated postnatal maturation, abnormal glomerular morphology and podocyte depletion are likely complex and multifaceted. scRNAseq performed in the rat model of preterm birth revealed 20 distinct clusters and 12 different cell types in preterm rats and full-term rats with significant differences in gene expressions (108). These candidate genes and pathways may provide targets to improve kidney health in preterm infants. In a study by Cwiek et al. kidneys of the preterm mice exhibited decreased proportions of endothelial cells and a lower expression of genes promoting angiogenesis in comparison with the term group. Similarly, in the lamb model of preterm delivery a reduction in glomerular capillary growth/length and glomerular filtration surface area has been observed, possibly contributing to the increased predisposition to hypertension (109, 110). Cwiek et al. also found alterations in the vitamin metabolism as well as altered nephron progenitor subpopulations, early SIX2 depletion, and altered JAG1 expression in the nephrogenic zone, suggesting a premature differentiation of nephron progenitor cells (104).

## 7 Clinical management and follow-up

There are currently no guidelines on how to identify, follow-up and treat infants born prematurely who are at increased risk of developing AKI and/or CKD. Moreover, any recommendation for population-based screening must be tempered by the acknowledgment that there is no specific therapy to arrest the progression of CKD. Nevertheless, early detection of kidney adverse outcomes raises awareness for cardiovascular risk reduction including blood pressure control as well as treatment of proteinuria, avoidance of potential nephrotoxins and adjustment of drug dosing, all of which have been proven to slow the progression of CKD.

In 2017, the Low Birth Weight and Nephron Number Working Group released a consensus document aimed to address the developmental programming of hypertension and CKD (111). This document emerged from a workshop among experts in the field of obstetrics, neonatology, and nephrology (112). It includes recommendations to decrease the risk of preterm delivery as well as recommendations that aim to prevent CKD and other related noncommunicable diseases in individuals born preterm. The latter will be the focus of this section. Importantly, this clinical guidance document is based on expert opinion; no evidence-based recommendations are currently available.

Firstly, documentation of GA at birth and neonatal course should be an integral part of an individual’s health record. Infants should be weighed at birth, GA determined, and these facts registered. Moreover, in the early postnatal period, preterm birth should be recognized as a risk factor for neonatal AKI by neonatologists. Every effort to prevent AKI should be made (optimization of fluid management, avoiding the use of nephrotoxins, attention for nutritional status…) and in case AKI occurs, this should be documented in the discharge report. It was previously reported that only 13.5% of infants with neonatal AKI had it listed on their neonatal intensive care unit (NICU) discharge summaries (113). The working group further proposed that infants who experienced neonatal AKI, especially if preterm, require life-long follow-up of blood pressure and renal function. Very premature children (< 32 weeks GA) or children with AKI postnatally should be screened initially at 6 months and not later than 1 year of age with a serum creatinine/cystatine C measurement, a urinary albumin/creatinine ratio, a blood pressure measurement and, if feasible, a baseline renal ultrasound to detect small kidneys, renal asymmetries, or structural alterations. Other preterm infants should have a first screening not later than 3 years of age. Thereafter, screenings can be performed at planned checks of child health status, medical visits, or at 2-year intervals throughout school years. Through these screenings, kidney dysfunction can be detected based on the presence of microalbuminuria, systemic hypertension, reduced GFR and/or reduced renal volume. Screening should be integrated with other health activities if possible, to avoid labelling these children as “sick.” Families should be educated about healthy lifestyle strategies to minimize obesity and malnutrition. Rapid catch-up growth should be avoided to prevent obesity-associated exacerbation of the renal risk (114). From age 18 years onwards, monitoring of blood pressure, BMI, serum creatinine and urine analysis biannually until 40 years of age, and thereafter at yearly intervals is recommended. The periodicity of these tests should be adjusted according to the results or the appearance of comorbidities.

Caution must be taken in candidates for living kidney donation as recent studies have suggested that some living donors may be at increased risk of ESRD (115). Preterm birth and/or low nephron endowment could be a potential modifier of this risk. Questioning about birth circumstances should be routine in all potential donors. Potential donors who were born preterm should preferably not be accepted for donation if there is any proteinuria, elevation of blood pressure, diabetes or a BMI >25 kg/m^2^, and accepted donors should be closely followed, ideally by a nephrologist life-long.

## 8 Future directions

Essential knowledge gaps remain that are important to address in order to fully understand the renal consequences of preterm birth. These include (1) a deeper understanding of the (molecular) mechanisms underlying human kidney development, in particular the processes regulating nephron endowment and synchronous cessation of nephrogenesis; (2) mechanistic studies on how preterm birth and its associated pre-, peri-and postnatal factors cause abnormal and immature nephrogenesis; (3) a better understanding of the nature and duration of postnatal nephrogenesis after preterm birth; (4) confirmation or disproof of a reduced nephron endowment in the kidneys of infants born prematurely; (5) improved identification of factors most associated with CKD in former preterm infants, including potential sex differences; (6) mechanistic studies formally explaining the link between prematurity-associated impaired nephrogenesis and CKD later in life; and (7) the development of non-invasive, *in vivo* methods with which to assess renal functional capacity and identify at-risk individuals.

Currently, human kidney development is an area of intense study characterized by extraordinary complexity. The current knowledge continues to expand and is being propelled by the emergence of (spatial) omics. So far, a certain degree of heterogeneity between individuals has been recognized by the field, including heterogeneity in the timing of cessation of kidney development, in the numbers of nephrons and in cell numbers per nephron segment (e.g., 431–746 podocytes per adult glomerulus) (116). Unraveling the origins of these variations is likely to have an important impact on the field. For instance, Yermalovich et al. showed that the Lin28b/*let*-7 axis controls the duration of kidney development in mice and that suppression of *let-7* could prolong nephrogenesis and enhance kidney function (117). Laboratory-based studies and experimental models are necessary to understand the interplay of the myriad of factors (genetic and epigenetic modifiers, nutrition, medication…) that contribute to the course of kidney development and how this course is altered in preterm infants pre-and postnatally. This research could have the potential to identify strategies to rescue suboptimal nephrogenesis and augment nephron endowment in the kidneys of infants born preterm.

Follow-up of birth cohorts from early childhood up until late adulthood are necessary to improve our understanding of the relationship between prematurity and renal complications. The study by Gjerde et al., (101) showing that only subjects with at least two of the three risk factors LBW, SGA and preterm birth, had an increased risk of ESRD underlines the importance of robust epidemiological data that consider the distinction between these subcategories. Avoiding the LBW umbrella in future studies will allow identification of who is actually affected and permit specific monitoring programs restricted to risk patients instead of follow-up of large groups. In addition, sex hormones could play a protective role in one subcategory but not in the other, so that sex-specific effects could be missed in the overarching preterm group. Indeed, Gjerde et al. found a relation between preterm birth and ESRD in males but not in females; this difference was not revealed in the LBW or SGA group. Clearly, more longitudinal studies in childhood, adolescence and adulthood are needed to investigate this association in several populations and to address the potential cost versus benefit of such follow-up.

Finally, new methods to assess nephron number non-invasively in living individuals are urgently needed to guide both scientific inquiry and clinical care. Importantly, non-invasive nephron counting in living persons would also allow us to perform longitudinal studies determining the nephron number required for maintenance of long-term renal function. This is currently unknown and could potentially be sex-and race-dependent. Therefore, novel methods suitable for small tissue samples, and innovative approaches that permit non-invasive measurements of functional nephrons are of great interest. In experimental animals, cationic ferritin has been used safely to label the glomerular basement membrane, allowing an accurate count of glomeruli using MRI (48).

## 9 Conclusion

With the successes of neonatal care, the majority of premature infants are now surviving into adulthood. Growing evidence from observational studies and animal models demonstrate that these infants do not follow the normal trajectory of kidney development and are therefore at increased risk of kidney dysfunction later in life. This risk increases with lower GA at birth and the effect might be even stronger in infants with concurrent IUGR and SGA. Moreover, the increased risk of AKI in preterm neonates acts as a positive effect modifier in the relationship of prematurity and CKD. The exact pathophysiological mechanisms underlying the risk of CKD are currently incompletely understood and major hypotheses include a decrease in nephron endowment, podocyte depletion and vascular damage. Currently, there is no consensus on the follow-up of these patients and treatment and preventive strategies are missing. Future research including epidemiological studies, experimental, mechanistic research, and technological advances to assess nephron numbers non-invasively is clearly needed to address important knowledge gaps on the causality between preterm birth and kidney disease. Ultimately, this knowledge would inform interventions to rescue kidney development and improve kidney health in these susceptible individuals.

## Author contributions

SD: Writing – original draft, Writing – review & editing. MR: Writing – original draft. TF: Writing – original draft. LvdH: Writing – review & editing, Supervision. EL: Supervision, Writing – review & editing. FA: Supervision, Writing – original draft, Writing – review & editing.

## Glossary

**Table:** 

AGA	Appropriate for gestational age
AKI	Acute kidney injury
BMI	Body Mass Index
CA	Chronological age
CI	Confidence interval
CKD	Chronic kidney disease
CM	Cap mesenchyme
CSB	Comma-shaped body
ELBW	Extremely low birth weight
ELGAN	Extremely low gestational age neonates
ESRD	End-stage renal disease
GA	Gestational age
eGFR	estimated Glomerular filtration rate
HR	Hazard ratio
IUGR	Intra-uterine growth restriction
LBW	Low birth weight
MET	Mesenchymal-to-epithelial transition
MM	Metanephric mesenchyme
NICU	Neonatal intensive care unit
NSAID	Non-steroidal anti-inflammatory drugs
OR	Odds ratio
PMA	Postmenstrual age
PPROM	Preterm prelabor rupture of the membranes
PTA	Pretubular aggregate
RAAS	Renin-angiotensin-aldosterone system
RGC	Ray Glomerular Counting
RR	Relative risk
RV	Renal vesicle<
SSB	S-shaped body
scRNAseq	Single cell RNA sequencing
SCr	Serumcreatinine
SGA	Small for gestational age
UB	Ureteric bud
