# Supplementary figures and images for: Impact of preterm birth on kidney health and development
Source: Front Med (Lausanne). 2024 Mar 27;11:1363097. doi: 10.3389/fmed.2024.1363097 (PMC11004308; doi:10.3389/fmed.2024.1363097)

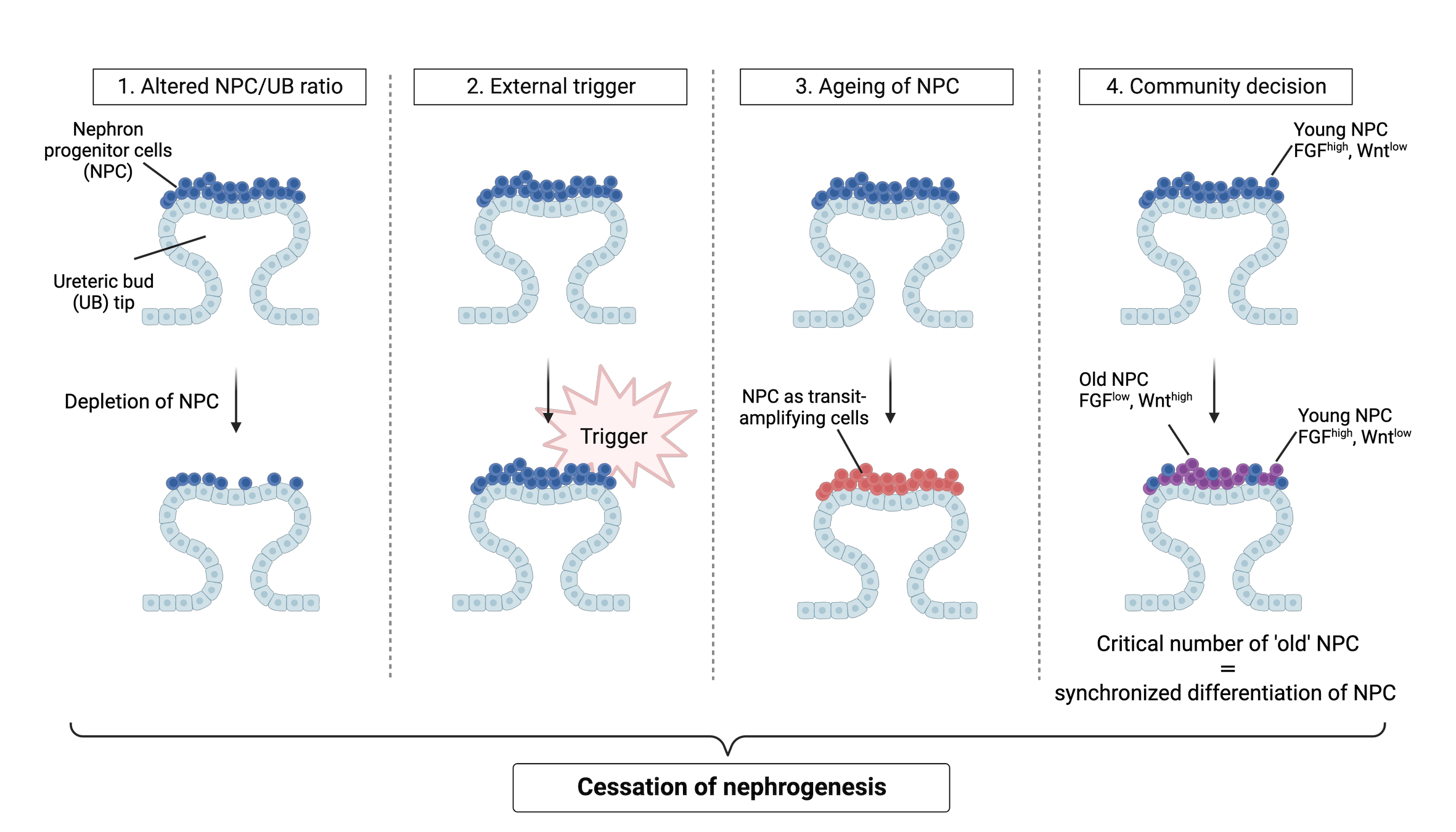

Supplement: Supplementary file 1 [file Data_Sheet_1.ZIP › Figures/Figure 2.tiff]

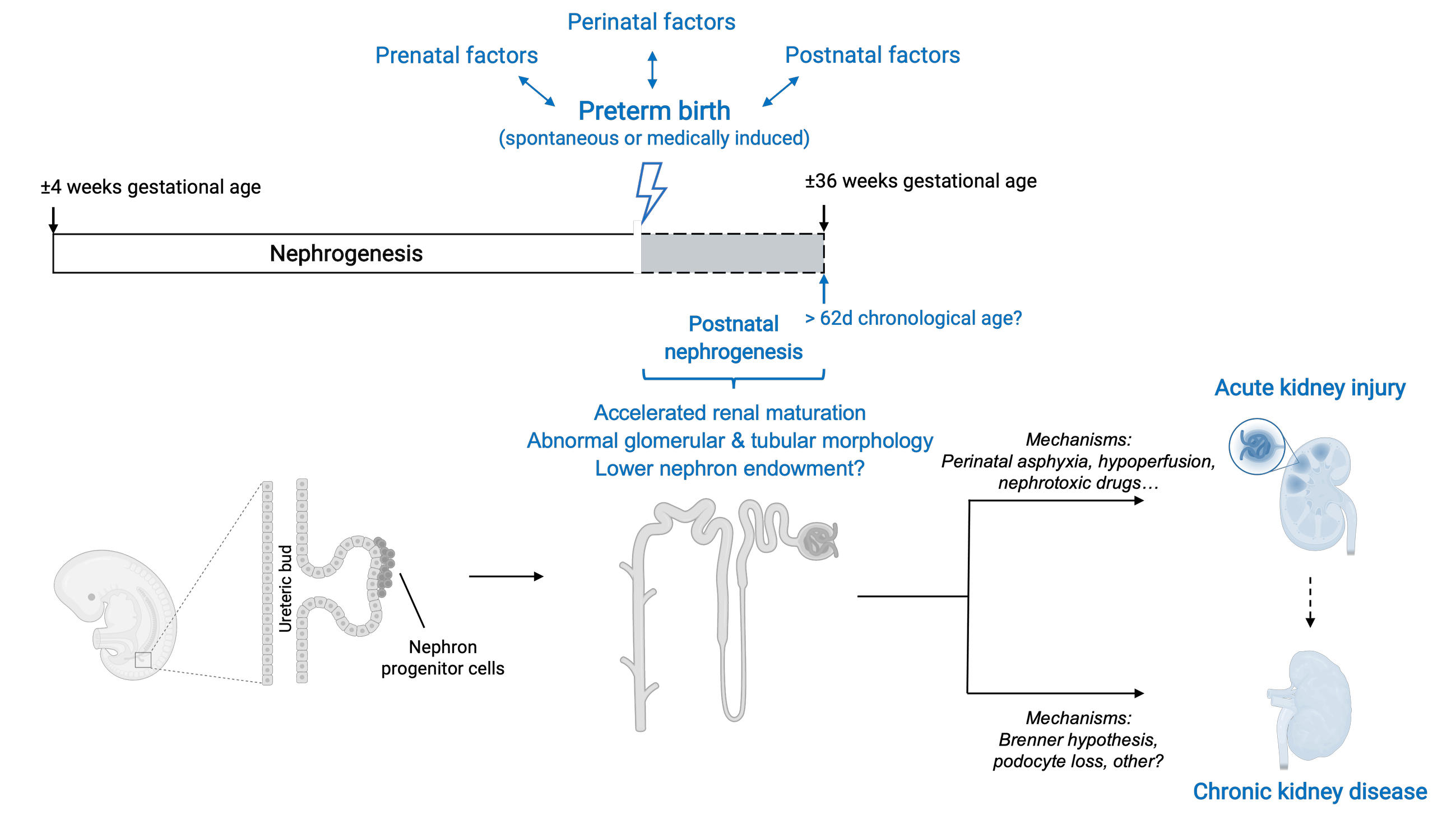

Supplement: Supplementary file 1 [file Data_Sheet_1.ZIP › Figures/Figure 3.tiff]

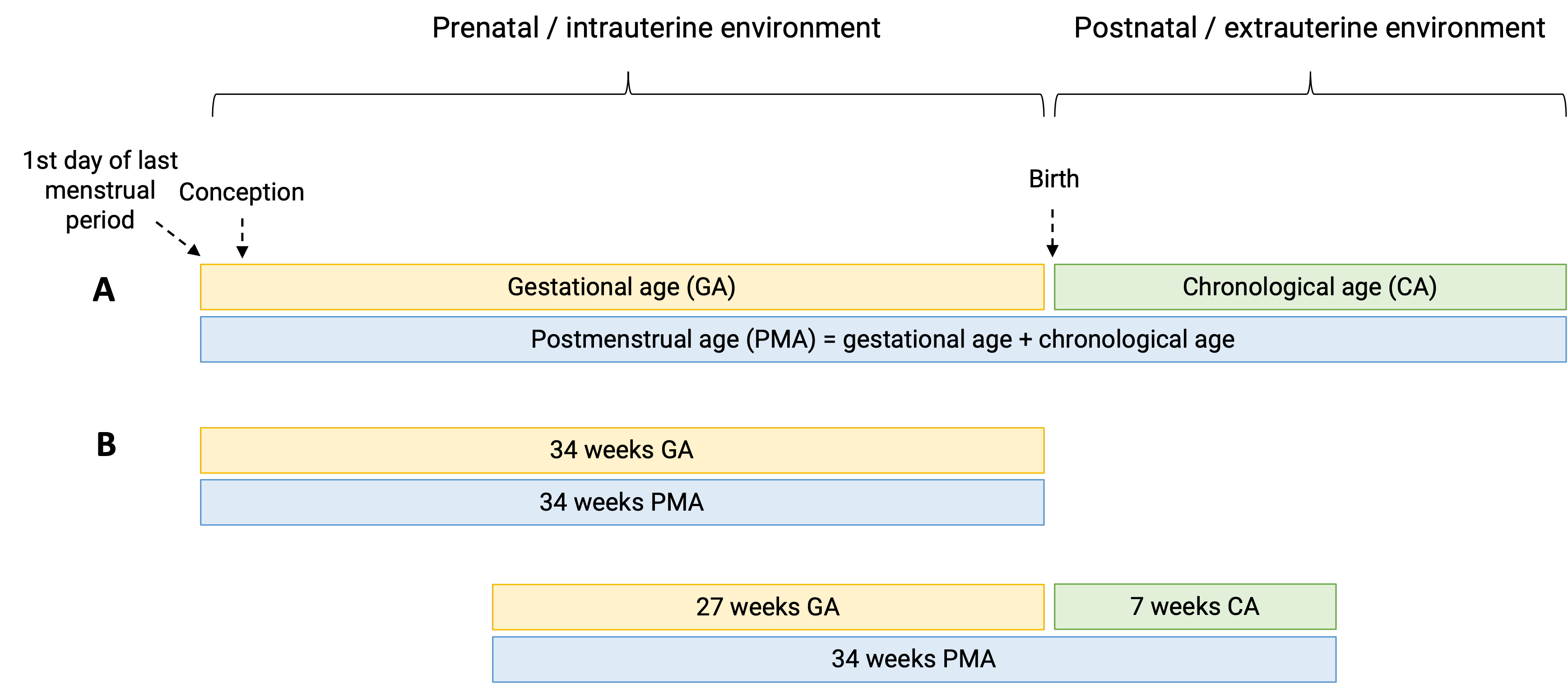

Supplement: Supplementary file 1 [file Data_Sheet_1.ZIP › Figures/Figure 1.tiff]
